# Urban built environment and its impact on university students’ loneliness: a mechanistic study

**DOI:** 10.3389/fpubh.2025.1514820

**Published:** 2025-03-13

**Authors:** Shuguang Deng, Jinhong Su, Heping Yang, Jinlong Liang, Shuyan Zhu

**Affiliations:** ^1^School of Geographic Sciences and Planning, Nanning Normal University, Nanning, China; ^2^Department of Neurology, Guangxi Zhuang Autonomous Region Nationalities Hospital, Nanning, China

**Keywords:** built environment, university students’ loneliness, spearman correlation, Geodetector, ridge regression model

## Abstract

**Introduction:**

With the acceleration of urbanization and social changes, loneliness among university students is becoming increasingly common. The urban built environment is closely related to loneliness. This study explores the impact of the urban built environment on the loneliness of university students from multiple built environment elements such as road network density, land use mix, and service facilities. It is of great significance to optimize urban planning and improve the mental health of university students.

**Methods:**

Based on questionnaire data and point interest data of various facilities, Spearman correlation analysis, Ridge regression model and geographic detector were used to explore the impact mechanism of urban built environment on loneliness of university students.

**Results:**

The study shows that loneliness is widespread and relatively severe among university students. The urban built environment is closely related to university students’ loneliness: the number of catering, transportation, tourist attractions, leisure and entertainment, healthcare, and sports facilities, as well as road network density, are significantly negatively correlated with loneliness, while the number of shopping facilities and land use mix are positively correlated with loneliness. Geographic detector analysis shows that tourist attractions, health care facilities and land mix have a significant impact on university students’ loneliness, and the interaction of multidimensional factors significantly improves the explanatory power of loneliness.

**Conclusion:**

To alleviate loneliness among university students, interventions should be approached from the perspective of urban planning and management. Firstly, it is essential to improve leisure, landscape, transportation, healthcare, and fitness facilities, enhancing their accessibility to foster social interactions. Secondly, increasing the availability of socially-oriented public spaces, such as student activity centers, community squares, and shared learning spaces, can strengthen interaction and communication. Additionally, policymakers should optimize the layout of urban transportation networks to encourage students to use public transit. Urban planners can support active transportation modes, such as walking and cycling, by rationally allocating road space. Lastly, the strategic placement of green and open spaces, such as parks and squares, should be prioritized to enhance access to natural environments, promote social activities, and mitigate feelings of loneliness.

## 1 Introduction

Loneliness, as a mental health issue, exerts a profound impact on individual well-being. Loneliness is a subjective and negative experience associated with insufficient social interactions or a lack of close relationships (1). University students, as a distinct group, encounter numerous challenges and pressures, leading to increasingly prominent mental health issues. The pressures of academics, social life, emotions, and environmental adaptation make university students susceptible to loneliness (2, 3). Prolonged or severe loneliness not only triggers emotional disorders and diminishes mental health but may also lead to extreme behaviors like suicide, posing threats to students’ growth and development (4). The Healthy Cities initiative, proposed by the World Health Organization in 2020, aims to promote the improvement of existing cities and the creation of quality living environments for all, enhancing well-being through health-promoting preventive measures rather than focusing solely on the treatment of disease (5). Thus, proposing mental health-promoting interventions for the environment from an urban planning perspective is an important way to prevent and reduce loneliness among university students.

The study of environment and psychology started early. In early individual studies, theories such as the Health Belief Model, Planned Behavior Theory, Social Cognitive Theory, and Stage Model of Behavioral Change from the fields of social cognitive psychology and behaviorism were used to explain how the environment affects specific behaviors such as learning and health behavior by affecting people’s psychology (6). In the late 1990s, James Sallis and others proposed the Ecological Model of Active Living, and later developed the Social-Ecological Health Model and the Healthy City Model on this basis, further clarifying the role of the built environment on people’s health behaviors and psychological states to varying degrees (7). The Social-Ecological Health Model provides a deep understanding and a comprehensive analytical framework for studying the impact of the urban built environment on mental health. From small groups and organizations to larger groups, it does not focus solely on individuals or the population, but combines multi-level analysis and different research methods and means (such as physical examinations, questionnaires, behavioral observations, environmental records, and epidemiological analysis) to assess the health of the environment and a good environment (8).

The relationship between the urban built environment and health has been a scholarly focus since 1999, with studies increasing annually, particularly since 2012, when mental health became a key subfield in this area (9). The urban built environment is a potential determinant of health and health inequalities, with mental health being one of the most significantly impacted health indicators by urban structure (6). Research indicates that urban morphology (10), land use diversity, street connectivity, and layout are closely linked to mental health (11–14). Hematian and Ranjba (15) found that land use diversity, transportation accessibility, walkability, and air quality are closely associated with mental health. Existing studies have shown that exposure to green spaces helps alleviate negative emotions and enhance mental health (16, 17). Research by Taylor et al. (18), Pasanen et al. (19), Markevych et al. (20), and Beyer et al. (21) has consistently shown that urban green spaces help reduce negative emotions, mitigate symptoms of depression and anxiety, and enhance mental health. Transportation accessibility and the spatial distribution of service facilities likewise have a significant impact on mental health. Evans (22) found that accessibility to public transportation is directly correlated with mental health. The proximity and diversity of infrastructure significantly boost mental health, and a well-planned layout of service facilities has a similarly positive effect (23–25). Research indicates that the urban built environment influences mental health through subjective perception, physical activity, and social interaction (26).

Currently, research on the impact of the urban built environment on loneliness is relatively limited. Existing studies primarily focus on the relationship between residents’ satisfaction with the built environment and community facilities and their feelings of loneliness. Research indicates that residents living in highly urbanized or impoverished communities with lower satisfaction regarding community quality exhibit a significantly higher prevalence of loneliness (27). In impoverished communities, the frequency or variety of facility usage is also negatively correlated with loneliness, meaning that higher facility usage is associated with lower levels of loneliness (28). Additionally, beyond satisfaction with community and its facilities, the adoption of diverse transportation modes (such as cycling, driving, and public transit) has been shown to significantly reduce feelings of loneliness (29). Furthermore, studies have found that built environments with high walkability can help alleviate loneliness among older adults (30), while improved accessibility to activity destinations and increased physical activity positively contribute to reducing loneliness. For younger individuals, built environment characteristics such as destination accessibility and location type influence their feelings of loneliness during daily activities, and commuting methods that promote physical activity, such as walking or cycling, may significantly alleviate emotional loneliness (31). Building density, green spaces, and recreational facilities are also closely associated with loneliness. Research suggests that residential density and green spaces can indirectly influence loneliness, while the availability of recreational services indirectly affects loneliness by promoting leisure and physical activities (32). Related studies also indicate that residents living in communities with more green spaces report lower levels of loneliness (33), whereas limited exposure to green environments may indirectly increase residents’ feelings of loneliness (34). Moreover, research has found that transit-oriented public space designs based on railways and buses can enhance the accessibility of urban environments, foster social interactions, and thereby reduce social loneliness (35).

Although studies on the relationship between the urban built environment and mental health have been growing in recent years, significant limitations remain. First, most existing studies focus on the mental health and loneliness of the older adult, with little attention paid to university students as a distinct group, thereby neglecting the unique pressures on their mental health and loneliness. Second, most research employs methods like Structural Equation Modeling, Multiple Linear Regression Model, surveys, and Ordinary Least Squares regression to study the impact of the built environment on the mental health of the older adult (36, 37) and different age groups (38, 39), but rarely integrates Geographic Information Systems and spatial analysis to uncover the underlying mechanisms. In addition, most studies on loneliness among university students are based on a psychological perspective, exploring its relationship with factors such as mobile phone dependence (40, 41), social support (42, 43), influencing factors of loneliness (2) and mediating effects (44–46), while ignoring the impact of the external physical environment on loneliness.

Therefore, based on the socio-ecological health model, we integrate the perspectives of geography and psychology. Our study employs methods including Spearman correlation analysis, the Ridge regression models, and geographical detectors. The aim is to reveal the impact mechanism of the urban built environment on university students’ loneliness, identify key environmental factors, and provide scientific evidence for urban planners, policymakers, and relevant government departments to optimize the urban environment and reduce university students’ loneliness. This will also provide valuable references for future related research. Our study fills the research gap regarding the impact of the urban built environment on university students’ loneliness, broadens the perspective of loneliness research, and offers specific intervention strategies to optimize urban planning and enhance students’ mental health.

## 2 Data and methods

### 2.1 Research hypotheses and research objectives

Loneliness among University students has become a widely recognized social and psychological health issue. As an external environmental factor, the urban built environment may have a significant impact on loneliness among university students (47). Therefore, our study aims to explore the specific influence of the urban built environment (such as public facilities, land-use patterns, road network density, etc.) on loneliness among university students. The central question of this study is: Which elements of the urban built environment are significantly associated with loneliness among university students?

Based on the research questions, we propose the following hypothesis:

Hypothesis 1: Elements of the urban built environment, such as public service facilities, land-use mix, road network density, and parks and squares, are negatively correlated with loneliness among university students. That is, the higher the density or accessibility of these facilities, the lower the level of loneliness among university students.

Hypothesis 2: Urban built environment elements have no significant impact on university students’ loneliness.

Our primary objective is to reveal the statistical associations between urban built environment characteristics and university students’ loneliness, as well as to identify the key environmental factors that have a significant influence on loneliness. The secondary objective is to compare the strength of the impact that different urban built environment factors have on loneliness, and to identify the most influential key factors. Furthermore, our study aims to provide scientific evidence for urban planners and policymakers to optimize the urban built environment and alleviate loneliness among university students.

### 2.2 Study area and data

As the capital of Guangxi Zhuang Autonomous Region, Nanning has developed rapidly, with a fast-paced lifestyle and high levels of competition that may increase university students’ psychological stress and loneliness. In recent years, Chongzuo has seen a gradual increase in educational resources, accompanied by rising student and competition pressures, which may contribute to higher levels of loneliness among university students. Therefore, we selected universities in the urban centers and suburban areas of Nanning and Chongzuo as case studies to compare the impact of different geographical locations on students’ mental health, ensuring the study’s representativeness. Taking into account geographical diversity, school types, campus sizes, and data availability, we ultimately selected students from 5 universities across 6 campuses in Nanning and Chongzuo, including Wuming Campus and Mingxiu Campus of Nanning Normal University, Guangxi Arts University, Guangxi University of Finance and Economics, Guangxi University, and Guilin University of Technology’s Nanning campus. We conducted a questionnaire survey among students from selected universities using a random sampling method between September and December 2023. A total of 330 valid samples were collected, and after removing missing values, 297 valid samples were ultimately obtained.

The urban built environment helps alleviate loneliness by providing a comfortable living environment, fostering social interactions and a sense of belonging, and offering abundant recreational facilities to meet the needs of university students. Most existing research explores the relationship between the urban built environment and mental health through the “5D” model (density, diversity, design, public transport accessibility, and destination accessibility), using indicators such as floor area ratio, building density, land use mix, road network density, and accessibility of parks and everyday facilities (6, 48, 49). Based on prior research, data availability, and the 5D model, the selected urban built environment indicators include the number of Points of Interests (POIs) (covering seven categories such as dining, shopping, tourism, healthcare, transportation, sports, and entertainment) and their total count, land use mix, and road network density. Land use mix refers to the degree of mixing of different types of land use within a specific area, reflecting the diversity and complexity of land use. In this study, land use mix is calculated using point of interest (POI) data and the entropy index. The entropy index is a commonly used method for measuring land use mix. It reflects the diversity of land use by calculating the uniformity of the distribution of different land use types within a given area. A higher entropy index indicates greater diversity in land use types, while a lower entropy index indicates more homogeneous land use. Built environment data, including road network density and POI data, were sourced from open platforms like Google Maps, Baidu Maps, Gaode Map, and Open Street Map (OSM). A 1,000-meter radius is generally considered a 15-min walkable living area, encompassing key facilities for students’ daily needs. Hence, relevant indicators were extracted within a 1,000-meter buffer extending outward from the school boundaries. To objectively measure loneliness among university students, we used the University of California, Los Angeles Loneliness Scale (UCLA) Loneliness Scale developed by Russell et al. (50), which has proven to have high reliability and validity in accurately assessing individual levels of loneliness.

### 2.3 Methods

**Figure 1:**
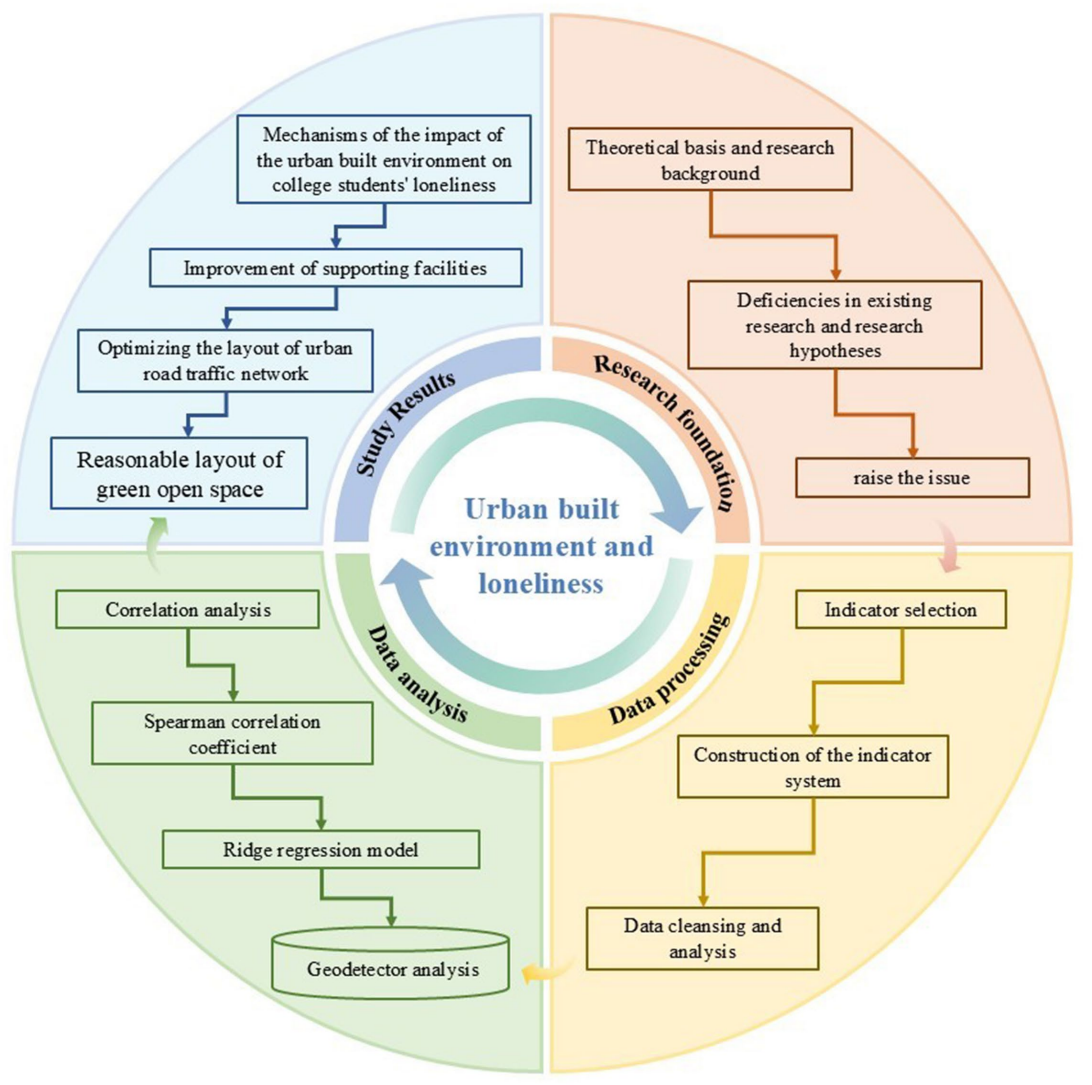
Research framework.

Figure 1 presents the overall theoretical framework for the study on “The Impact of the Urban Built Environment on University Students’ Loneliness.” The central part of the figure represents the core topic of the research: the impact mechanism of the urban built environment on university students’ loneliness. The entire framework revolves around this core topic and is divided into four main research directions:

Research background and problem formulation: the research background section systematically reviews the existing research findings on the impact of the urban built environment on loneliness and mental health through keyword and topic screening of relevant literature. This process clarifies the theoretical basis and background of the research, and based on this, proposes theoretical hypotheses and research questions, laying a solid academic foundation for the subsequent research.

Construction of the indicator system and data acquisition: our study first constructed an indicator system to measure the impact of the urban built environment on loneliness by referencing existing research findings. Subsequently, relevant data on the urban built environment were collected, and data cleaning and processing were conducted using SPSSPRO and ArcGIS software to ensure data quality and reliability.

Data analysis and model construction: based on questionnaire data and point of interest (POI) data, we employed Spearman correlation analysis in SPSSPRO to explore the linear relationship between built environment elements and loneliness among university students. This analysis identified indicators that significantly influence loneliness under a simple linear framework. Subsequently, we used Ridge regression models to further investigate the impact of the built environment on loneliness. Finally, Geodetector software was utilized to analyze key factors influencing loneliness and their interactions.

Conclusions and recommendations: based on the correlation analysis results between the urban built environment and loneliness among university students, strategies and recommendations were proposed to optimize the urban built environment and alleviate loneliness among university students.

#### 2.3.1 Spearman correlation coefficient

The Spearman correlation coefficient is a classic statistical method used to measure the correlation between ranked variables, serving as an extension of Pearson’s correlation coefficient. Spearman correlation ranks variables and is suitable for analyzing non-normal distributions or non-linear relationships, making it appropriate for this study as the observed indicators follow a non-normal distribution. See Equation 1 for details on the coefficient ranges from −1 to 1, with negative values indicating a negative correlation, positive values indicating a positive correlation, and 0 indicating no correlation. The formula is expressed as:


(1) $$\rho =1-\frac{6\sum {d_{i}}^{2}}{n\left(n^{2}-1\right)}$$

Where *ρ* is the Spearman correlation coefficient; *n* is the sample size; *d_i_^2^* represents the squared rank difference for each pair of observations, and *Σd_i_^2^* is the sum of squared rank differences for all samples.

The Spearman correlation coefficient excels in handling continuous variables and offers clear interpretations of correlation, making it ideal for initial screening of related variables and laying the groundwork for further regression analysis. We used the Spearman correlation coefficient to analyze the relationship between urban built environment indicators and university students’ loneliness, effectively capturing the linear correlation between the two and offering a preliminary evaluation of the correlation between the built environment and loneliness.

#### 2.3.2 Ridge regression model

Ridge regression introduces a regularization term to the ordinary least squares (OLS) regression to prevent overfitting caused by multicollinearity. Ridge regression not only effectively addresses multicollinearity but also maintains lower variance, providing more stable parameter estimates, making it particularly suitable for this study, which involves numerous feature variables and potential multicollinearity. With the numerous urban built environment indicators and potential multicollinearity in this study, the Ridge regression model enables a more accurate assessment of the impact of each indicator on university students’ loneliness, eliminating the influence of other variables and facilitating a deeper understanding of how the built environment affects loneliness. See Equation 2 for details of the Ridge regression model is:


(2) $$\hat{\beta }={\left(X^{T}X+\lambda I\right)}^{-1}X^{T}y$$

Here, $\hat{\beta }$ is the vector of regression coefficients in the Ridge regression model, representing the weight assigned to each independent variable by the model. *X* is the design matrix (of size n∗p ), where *n* is the sample size and *p* is the number of independent variables. *Xᵀ* is the transpose of the design matrix *X* (of size p∗n ). *y* is the vector of dependent variables (of size n∗1 ). *λ* is the regularization parameter (*λ* ≥ 0), which controls the strength of regularization. *I* is the identity matrix (of size p∗p ), used to ensure the invertibility of the matrix ( $X^{T}X+\lambda I$ ).

#### 2.3.3 Geodetector

The Geodetector is a statistical method based on spatial analysis that identifies the relationships between spatial variables and their spatial heterogeneity. Compared with traditional methods, the Geodetector can reveal the influence of built environment elements on loneliness across different regions and identify key factors. The Geodetector is capable of handling both discrete and continuous variables without relying on a specific regression form, making it flexible in revealing spatial heterogeneity and quantitatively assessing the influence of variables, which is particularly important for the complex variable relationships in this study. See Equation 3 for details of the Geodetector is:


(3) $$q=1-\frac{1}{n\sigma^{2}}\overset{L}{\underset{h}{\sum }}n_{h}\sigma_{x}^{2}$$

where *q* represents the explanatory power of the factor, *n* is the total number of samples in the study area, *n_h_* is the number of samples in layer h, L is the stratification of the dependent or independent variable, and *σ^2^* is the overall variance of the study area. In the Geodetector’s factor detection, the q value indicates the extent to which built environment factors influence university students’ loneliness. The q value ranges from [0, 1], with higher values indicating greater impact of the built environment factors on loneliness, and lower values indicating lesser impact. The *p*-value is a measure of statistical significance that assesses the credibility of the results. Smaller *p*-values indicate that the results are less likely to occur by chance, with *p*-values below 0.05 generally regarded as statistically significant. We used the Geodetector to analyze the relationship between urban built environment indicators and university students’ loneliness, enabling a quantitative assessment of how different factors influence loneliness, thus offering a more comprehensive understanding of the impact of built environment indicators on loneliness.

## 3 Results

### 3.1 Descriptive analysis of the sample

To gain a deeper understanding of loneliness levels among university students, we use the UCLA Loneliness Scale to measure individuals’ experiences of loneliness in social relationships. The scale consists of 20 items, using a four-point Likert scale. The total score is the sum of all item scores, with a higher total indicating a greater degree of loneliness. Additionally, to distinguish between different levels of loneliness, we classify the total loneliness scores of university students based on relevant literature. The grouping criteria are as follows: below 28 points represents low loneliness, 28–33 points represents below-average loneliness, 33–39 points represents moderate loneliness, 39–44 points represents above-average loneliness, and above 44 points represents high loneliness (51). Survey results showed that 97% of students experienced moderate to high levels of loneliness, indicating that loneliness is extremely widespread and severe among university students. Among them, 19% exhibited above-average loneliness, while 70% suffered from high loneliness. These data clearly reflect the prevalence and severity of loneliness among university students. An analysis of the five schools revealed that the average loneliness scores across all schools were above 44 points, indicating high loneliness. On an individual level, university students’ loneliness may be linked to personality traits, psychological resilience, and self-perception. Some students set overly high expectations or have distorted self-worth, which contributes to feelings of loneliness. Moreover, the fast-paced lifestyle, high levels of competition, and alienation of interpersonal relationships in modern society make it difficult for students to find confidants when dealing with academic or job-related pressures. While social media offers more communication channels, it often leads people to immerse themselves in the virtual world, neglecting real-life interactions, which further exacerbates loneliness. Therefore, examining the impact of the urban built environment on university students’ loneliness can guide urban planning in creating environments that promote social interaction, reduce loneliness, and improve mental health.

### 3.2 Spearman correlation coefficient analysis

University students’ loneliness was set as the dependent variable, with the urban built environment as the independent variable. The independent variables included the number of Points of Interests (in seven categories: dining, shopping, tourism, healthcare, transportation, sports, and entertainment), total facility count, land use mix, and road network density. If the two variables show consistent trends, there may be a relationship, which should be validated and quantified through data analysis (52). After testing the observed indicators, we found that most variables exhibited non-normal distributions, except for a few that followed a normal distribution. Therefore, Spearman correlation coefficients were used to analyze the relationship between the urban built environment and university students’ loneliness, initially identifying significant built environment variables related to loneliness and their corresponding coefficients (Table 1).

**Table 1:** Spearman correlation analysis results.

Indicator	Spearman correlation coefficient
Dining facilities	0.318 (0.000***)
Shopping facilities	0.318 (0.000***)
Transportation facilities	−0.725 (0.000***)
Tourist attractions	−0.776 (0.000***)
Leisure and entertainment facilities	−0.826 (0.000***)
Healthcare facilities	−0.873 (0.000***)
Sports and fitness facilities	−0.826 (0.000***)
Total number of service facilities	−1.000 (0.000***)
Road network density	−1.000 (0.000***)
Land use mix	−0.796 (0.000***)

***, **, and * represent significance levels at 1, 5, and 10%, respectively.

The study indicates a significant statistical relationship between loneliness and urban built environment indicators (*p* < 0. 01), suggesting that university students’ loneliness is correlated with various observed variables. However, different built environment factors have varying impacts on loneliness among university students. Within a 1,000-meter radius around the campus, the correlation coefficients for transportation, tourist attractions, recreational facilities, healthcare and fitness facilities, and total service facilities are negative and relatively large in magnitude, indicating a significant negative correlation with loneliness among university students (*p* < 0.01). This suggests that the more comprehensive the service facilities, the lower the level of loneliness among university students. The correlation coefficient for dining and shopping facilities is 0.318 (*p* < 0. 01), indicating that an increase in dining and shopping facilities does not alleviate loneliness among university students; in fact, it exacerbates their loneliness. Furthermore, loneliness among university students shows a significant negative correlation with road network density and land-use mix, indicating that the higher the road network density and land-use mix, the lower the level of loneliness.

### 3.3 Ridge regression analysis

Due to the high correlation between independent variables, the estimation of the least squares method will become unstable, resulting in the coefficients of the model becoming very large, and even error amplification, that is, multicollinearity. Ridge regression can reduce the coefficients of the model by adding regularization terms to the loss function, thereby alleviating the multicollinearity problem. Therefore, based on the Spearman correlation analysis, we use the Ridge regression model to further study the correlation between loneliness and urban built environment. Table 2 presents the results of the Ridge regression model, including the unstandardized coefficients (*B*), standardized coefficients (Beta), *t*-values, significance levels (*p*-values), and overall model fit (adjusted *R^2^* and *F* value). The constant term is 45.03, representing the predicted value of loneliness when all independent variables are zero. The Ridge regression results indicate that the *p*-value based on the F-test is less than 0.001, suggesting a significant regression relationship between the independent and dependent variables. Additionally, the model’s goodness of fit, R^2^, is 0.973, indicating that the model has a high explanatory power.

**Table 2:** Ridge regression model results.

K = 0.099	Unstandardized coefficients		Standardized coefficients	t	P	R^2^	Adjusted R^2^	F
	B	Standard error	Beta					
Constant	45.030	0.117	-	386.196	0.000***	0.973	0.971	915.959 (0.000***)
Dining facilities	0.000	0.000	0.039	8.636	0.000***			
Shopping facilities	0.000	0.000	−0.027	−4.705	0.000***			
Transportation facilities	−0.001	0.000	−0.135	−41.738	0.000***			
Tourist attractions	−0.007	0.000	−0.118	−32.789	0.000***			
Leisure and entertainment	−0.003	0.000	−0.158	−84.691	0.000***			
Healthcare facilities	−0.001	0.000	−0.144	−45.797	0.000***			
Sports and fitness facilities	−0.004	0.000	−0.155	−54.461	0.000***			
Total number of service facilities	0.000	0.000	−0.192	−73.768	0.000***			
Road network density	−0.040	0.001	−0.266	−48.426	0.000***			
Land use mix	4.121	0.182	0.258	22.675	0.000***			

***, **, and * represent significance levels at 1, 5, and 10%, respectively; K represents the minimum value at which the standardized regression coefficients for the independent variables stabilize.

Specifically, there is a significant correlation between the urban built environment indicators and loneliness among university students. The results show that the regression coefficient for dining and food facilities is 0.039, indicating a significant positive correlation with loneliness among university students (*p* < 0.001), consistent with the results of the Spearman correlation analysis. Apart from dining and food facilities, the regression coefficients for shopping and consumer facilities, transportation, tourist attractions, recreational facilities, healthcare, fitness facilities, total facility count, and road network density are all negative, indicating significant negative correlations with loneliness among university students. The enhancement of tourist attractions, dining, and leisure facilities directly influences students’ daily life experiences, providing more social opportunities and activity choices, resulting in positive emotions like happiness and satisfaction, which alleviate loneliness and anxiety, promoting mental health. The convenience of transportation facilities and the increase in road network density not only improve travel efficiency and convenience, but also make areas with convenient transportation more likely to attract crowds. The use of different modes of transportation (such as cars and public transportation) can also promote social activities (29) and reduce loneliness. The road network density affects the scope and frequency of daily travel. The road network density promotes the proximity of destinations, affects residents’ travel route selection, and promotes the improvement of adults’ physical activity levels (53). Higher road network connectivity and intersection density help slow down vehicle speeds, promote walking and social interaction (54), reduce loneliness, and improve mental health. In addition, the increase in transportation facilities and road network density also facilitates access to shopping, medical care, entertainment and other resources, and reduces psychological stress and loneliness by increasing physical activity.

The enhancement of healthcare and sports facilities offers health security for university students. Comprehensive healthcare facilities can alleviate the distress caused by mental health problems, reducing psychological stress and loneliness. The expansion of sports and fitness facilities offers more exercise opportunities for university students, improving their physical condition and positively influencing mental health, helping to relieve stress, maintain a positive mindset, and reduce loneliness. Research indicates that exercise has a positive impact on mood, and participating in physical activities can effectively reduce stress, general anxiety, social anxiety, and loneliness, among other mental health issues (55, 56). Furthermore, physical exercise can increase social opportunities, fulfill university students’ social needs, enhance interpersonal interactions and social skills, and reduce loneliness and other negative emotions (57).

The standardized coefficient for land-use mix is 0.258, indicating a significant positive impact on loneliness (*p* < 0.01). An increase in land use mix may contribute to regional congestion and noise due to high population density and diverse land use, which can reduce university students’ willingness to socialize, decrease social connections, and increase loneliness. In summary, the increase in dining, tourism, transportation, healthcare, sports and fitness facilities, along with road network density, plays a crucial role in reducing university students’ loneliness and enhancing mental health.

### 3.4 Geodetector results

The Geodetector factor detection results indicate that the impact of urban built environment indicators on university students’ loneliness is significant, with *q* values all above 0.870 (Table 3). The correlation between the three indicators of tourist attractions, Healthcare facilities and land use mixture and loneliness is extremely significant, with the *q* value close to 1 and the *p* value close to 0, indicating that they are closely related to loneliness and are highly statistically significant. However, although the *q*-values of restaurants, shopping, Leisure and entertainment facilities, transportation facilities, leisure and entertainment, the total number of service facilities and road network density are relatively high, the *p*-value is 1, indicating that their correlation with loneliness is not statistically significant and further verification is needed.

**Table 3:** Geodetector factor detection results.

Indicator factors	*q* value	*P* value
Dining facilities	0.870	1.000
Shopping facilities	0.920	1.000
Transportation facilities	0.920	1.000
Tourist attractions	0.999	0.000
Leisure and entertainment facilities	0.870	1.000
Healthcare facilities	0.999	0.000
Sports and fitness facilities	0.920	1.000
Total number of service facilities	0.920	1.000
Land use mix	0.999	0.000
Road network density	0.870	1.000

**Figure 2:**
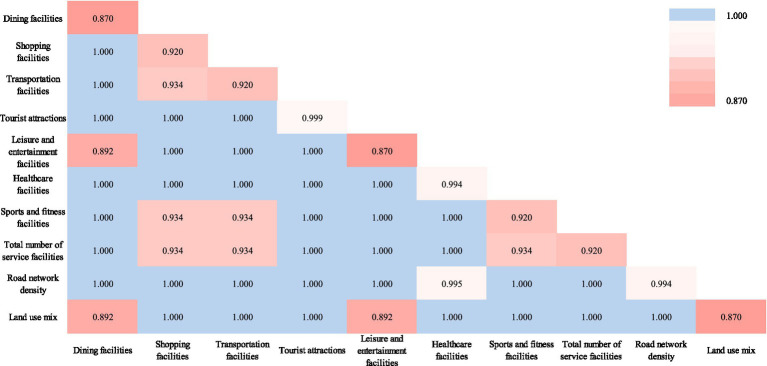
Driving factor interaction detection results.

From the results of the interactive detection of driving factors, we can see (Figure 2) that the interactions between the factors are significant. The interactions between factors such as catering and food facilities, shopping and consumption facilities, and transportation facilities and other factors are the most significant, especially the interactive explanatory power of catering and food facilities is the lowest at 0.87, and its explanatory power is significantly improved in combination with shopping and consumption, transportation facilities, and service facilities. For example, the combined interactive explanatory power of catering and food facilities and shopping and consumption facilities is 0.92, while the combination with transportation facilities is increased to 0.934, indicating that the joint effect of these factors can more effectively alleviate loneliness. Overall, the impact of a single facility on the loneliness of university students is relatively limited, while the synergistic interaction of multiple factors can significantly improve the explanatory power. This means that in urban planning, we should pay attention to the coordination and combination of multi-dimensional and multi-type facilities, especially the organic combination of infrastructure and public service facilities, so as to effectively improve the quality of life of university students and alleviate loneliness.

## 4 Discussion

Through the use of Spearman correlation analysis, Ridge regression models, and the Geodetector, we thoroughly examined the impact of the urban built environment on university students’ loneliness, confirming a significant correlation between the two. The study reveals that loneliness has become a widespread and serious issue among university students, significantly associated with several urban built environment factors. Specifically, the availability of dining, transportation, tourist attractions, leisure and entertainment, healthcare, and sports and fitness facilities are nificantly negatively correlated with university students’ loneliness, suggesting that the more comprehensive these service facilities are, the lower the level of loneliness. The negative correlation between dining facilities and loneliness may be attributed to the social interaction opportunities they provide. Furthermore, an efficient transportation system enables students to easily access various destinations such as employment, socializing, recreation, and shopping (58), thereby reducing their sense of isolation. Tourist attractions and recreational facilities offer venues for social interaction and leisure activities. Studies have shown that engaging in leisure activities significantly improves mental health and effectively reduces loneliness (59).

The accessibility of healthcare services ensures that students can obtain necessary support, thereby alleviating stressors that contribute to loneliness. The negative correlation between the availability of fitness facilities and loneliness may stem from the dual benefits of physical activity on mental health. On the one hand, physical exercise can directly improve mental health and effectively reduce loneliness (2); on the other hand, fitness venues provide students with opportunities for social interaction, further alleviating loneliness. These findings are consistent with previous research, indicating that the presence, availability, and quality of facilities are important factors influencing loneliness. Facilities can provide environments that foster social connections (52), and participation in social activities significantly reduces feelings of loneliness and depression (60). Through interactions with others, individuals can share their feelings and experiences, thereby alleviating psychological burdens and further reducing loneliness.

We also found that shopping facilities and land use mix are both positively correlated with university students’ loneliness, indicating that the increase in shopping facilities may exacerbate feelings of loneliness. This may be because university students tend to rely on shopping as emotional compensation when they feel lonely (61). Individuals who feel socially excluded may experience temporary happiness and satisfaction through impulsive consumption, believing that they have re-established connections with society (62), but the temporary satisfaction from shopping cannot alleviate deep-seated feelings of loneliness, and consumption comparisons (63) and a lack of effective social interactions (64) may further exacerbate loneliness.

Furthermore, our research also found that land use mix is positively correlated with university students’ loneliness. Areas with high land use mix typically integrate multiple functions (such as commercial, residential, and entertainment), and while this diversity improves convenience, it may also exacerbate social fragmentation. Highly mixed-use urban environments may weaken community cohesion, as the frequency of interactions between residents is relatively low, making it difficult to form stable social networks. Although areas with high land use mix provide a variety of activities and venues, they are often accompanied by high population density and frequent activities, which may lead to sensory overload and psychological stress. Sensory overload in the urban environment (such as noise and crowding) can exacerbate individuals’ psychological stress (65), thereby affecting their mental health. University students in such an environment may be more prone to feelings of fatigue and anxiety, and thus tend to isolate themselves, further exacerbating their loneliness.

Geodetector analysis results show that built environment elements such as tourist attractions, Healthcare facilities and land use mixture have the greatest impact on university students’ loneliness. Specifically, these factors may effectively alleviate university students’ loneliness by enhancing accessibility, increasing physical activity and social opportunities, and enriching daily life experiences. Although built environment indicators such as restaurants, shopping, Leisure and entertainment facilities, transportation facilities, leisure and entertainment, the total number of service facilities and road network density have a high correlation with loneliness, they do not reach a statistically significant level (*p* value is 1). The cause of this phenomenon may be due to the limited sample size or multicollinearity between the variables. Future research could expand the sample size or employ multivariate methods to further explore the potential impact of factors with lower significance on loneliness. In addition, the results of factor interactions show that the impact of a single facility on university students’ loneliness is relatively limited, while the synergistic interaction of multiple factors can significantly improve the explanatory power. Therefore, the key to optimizing the environment lies in the comprehensive coordination of various facilities, especially in the coordinated promotion of improving facility accessibility, resource diversity and health protection.

In urban design, providing people with an environment where they can access physical space, enjoy fresh air, feel safe, and promote social interaction can help reduce mental health problems and loneliness (66). Therefore, to reduce loneliness and improve the mental health of university students, proactive interventions in the built environment should be made from the perspective of urban planning and management, in order to optimize the environment and alleviate loneliness. First, ensure the diversity of supporting facilities and activity spaces around universities to meet the needs of modern urban students. By enhancing the variety and accessibility of leisure, landscape, transportation, healthcare, and exercise facilities, students’ social interactions can be promoted, which in turn reduces loneliness and enhances their mental health. Secondly, increase the provision of socially oriented public spaces. Research has shown that access to public spaces and the use of public facilities are associated with reduced loneliness (28). By planning more public spaces (67), such as student activity centers, community squares, and shared learning spaces, social interactions among university students can be effectively promoted, thereby alleviating loneliness. Studies indicate that factors such as road accessibility, the quantity, and diversity of service facilities have a significant positive impact on reducing loneliness among university students, suggesting that optimizing the urban built environment is an important strategy for alleviating loneliness and improving mental health. Therefore, it is essential to enhance the planning and construction of public service facilities, such as transportation, healthcare, and fitness facilities, to improve service quality and coverage, creating a more convenient and comfortable living environment. At the same time, policymakers should optimize the layout of urban road networks, enhance transportation convenience, and strengthen the public transportation system to encourage university students to choose public transportation. Research shows that compared to using private cars, individuals who use public transportation not only have more opportunities for social interaction (29), but also promote physical activities such as walking (68), which in turn increases their level of physical activity. Furthermore, the availability and accessibility of modes of transportation like walking, cycling, and public transit are key components of ensuring equitable access to public interaction opportunities (69). Additionally, urban planners can develop strategies to optimize road space allocation to support active transportation modes. For example, providing segregated and safe infrastructure, reducing the externalities of motor vehicles (70), and encouraging university students to adopt active commuting methods to reach their destinations can improve physical and mental health and reduce loneliness. Urban greening may be an important strategy at the community level to reduce the risk of loneliness (71). Reasonably planning parks, plazas, and other green spaces and open areas not only provides outdoor activity spaces for university students, allowing them to connect with nature and relax, but also creates a positive social environment that fosters social interaction (72, 73), thereby reducing loneliness.

Our study has the following limitations, which need to be addressed in future research. First, we only examined the correlation between the urban built environment and university students’ loneliness. Although it confirmed the impact of built environment factors on loneliness, it did not deeply analyze the specific mechanisms of this influence. Secondly, we did not sufficiently control for the influence of individual factors on the research results. Additionally, while the study primarily focused on the impact of the built environment on university students’ loneliness, it did not fully consider other potential risk factors such as mental health status, family background, academic pressure, and the quality of social networks, all of which may also have a significant impact on loneliness. Future research could take a more comprehensive approach by considering multiple factors to better understand the causes of loneliness and provide a theoretical basis for developing more targeted intervention measures. Moreover, the questionnaire design was not comprehensive enough, which may not fully reflect the impact of urban built environment factors on university students’ loneliness.

## 5 Conclusion

Our study aims to explore in depth the impact of urban built environment factors on university students’ loneliness. Using Spearman correlation analysis and Ridge regression models, we systematically examined the relationship between urban built environment factors and university students’ loneliness. The results show that service facilities such as dining, transportation, tourism, leisure and entertainment, healthcare, and sports and fitness, as well as road network density, are significantly negatively correlated with university students’ loneliness. However, the increase in shopping and consumer facilities and land use mix may exacerbate students’ loneliness to some extent. Geodetector analysis further revealed that tourist attractions, healthcare facilities, and land use mix have a significant impact on university students’ loneliness. These findings support the theory that service facilities such as dining, shopping and consumer facilities, transportation, tourist attractions, leisure and entertainment, healthcare, and sports and fitness, as well as road network density and land use mix, have a significant effect on university students’ loneliness. This provides important insights for urban planners and architects, suggesting that optimizing the built environment and design strategies can effectively reduce university students’ loneliness and improve their mental health. This study not only confirms the significant impact of urban built environment factors on university students’ loneliness but also provides empirical support for future urban planning and architectural design. It is recommended that urban planners and architects carefully consider the accessibility of service facilities and the rational layout of land use when planning and designing urban spaces to promote social interaction among university students and improve their mental health.

## Data Availability

The raw data supporting the conclusions of this article will be made available by the authors, without undue reservation.
